# Acute Pancreatitis Following Pharmacomechanical Thrombectomy: A Rare Complication

**DOI:** 10.7759/cureus.81895

**Published:** 2025-04-08

**Authors:** Natasha Aghtarafi, Emma J Whitehall, Hesham Abdelkader, Deepa R John, Mohamed Kasem

**Affiliations:** 1 Radiology, Ipswich Hospital, Ipswich, GBR; 2 Radiology, Colchester General Hospital, Colchester, GBR

**Keywords:** acute pancreatitis, angiojet, iliofemoral vein thrombosis, interventional radiology, may-thurner's syndrome, percutaneous thrombectomy

## Abstract

A 35-year-old female presented to the emergency department (ED) with a three-day history of left lower limb edema and discomfort. A venous duplex scan and computed tomography (CT) venogram showed a left iliofemoral deep vein thrombosis (DVT). The CT also demonstrated compression of the left common iliac vein by the right common iliac artery, suggesting May-Thurner syndrome.

The iliofemoral DVT was treated with pharmacomechanical thrombectomy (PMT), which resulted in successful revascularization of the left common iliac and femoral veins.

The following day the patient developed severe epigastric pain with raised amylase levels, leading to a diagnosis of acute interstitial pancreatitis, which was thought to be secondary to massive hemolysis.

Despite the general tolerability of mechanical thrombectomy, complications such as hemorrhage, pseudoaneurysm, and dissection may occur.  Acute pancreatitis post-thrombectomy is rare, with very few cases reported in the literature, primarily documented in patients with renal insufficiency and co-morbidities. Awareness of acute pancreatitis as a rare complication post-procedure is crucial, especially when abdominal pain is unexplained.

## Introduction

The treatment for venous thromboembolism (VTE) primarily involves anticoagulation, which aims to reduce the risk of clot propagation or distant embolism, but has little effect on the patient’s symptoms, such as pain [1]. Current National Institute for Health and Care Excellence (NICE) guidelines in the United Kingdom recommend at least three months of oral anticoagulation post-diagnosis of DVT, with a longer course of treatment if the event was unprovoked [2].  In the United Kingdom, the incidence of VTE is 0.1%, with approximately 20% of these cases manifesting as iliofemoral DVT [3]. Several known risk factors predispose individuals to iliofemoral DVT, including pregnancy, previous DVTs, invasive iliac venous procedures, and anatomical variants such as May-Thurner syndrome [4].   

Iliofemoral DVT is a severe type of venous thrombosis that affects the iliac and femoral veins and may extend into the inferior vena cava (IVC). This condition can cause complications such as pain, swelling, and skin changes if not managed promptly. Treatment solely with anticoagulation often results in poor outcomes [5], necessitating more aggressive treatment strategies than those used for other types of DVTs [6]. Advanced treatments, such as thrombectomy and catheter-directed thrombolysis, are frequently recommended over anticoagulation alone to prevent long-term complications like post-thrombotic syndrome. These interventions aim to rapidly clear the thrombus, restore venous patency, and reduce the likelihood of chronic symptoms [7].   

The AngioJet™ system is a type of peripheral pharmacomechanical thrombectomy (PMT) system by Boston Scientific, used to treat both venous and arterial thrombi, typically utilized for iliofemoral DVTs, and has a success rate of 94% [8]. The catheter emits a high-pressure jet of saline solution and thrombolytic agent that creates a localized area of low pressure at the tip, producing a Venturi (vacuum) effect that facilitates thrombus fragmentation and aspiration. This system is associated with good clinical outcomes and a reduced incidence of post-thrombotic syndrome [9]. Studies have shown that it offers a shorter treatment duration than catheter-directed thrombolysis [10].   

While highly effective in treating DVTs, the AngioJet™ PMT system has been associated with minor complications such as bradycardia and hemoglobinuria. More serious complications can include major hemorrhage and renal failure. There have also been a few case reports of acute interstitial pancreatitis in patients with pre-existing conditions such as renal failure [10-12].   

## Case presentation

A 35-year-old female presented to the emergency department (ED) with a three-day history of progressive swelling and pain in her left leg. Upon examination, the leg was significantly swollen, warm, and erythematous. Initial blood tests showed no abnormalities of note.  

Shortly before the onset of her symptoms, the patient had undergone a surgical evacuation of retained products of conception due to a miscarriage at 21 weeks gestation. She reported no significant past medical history or family history of DVT or thrombophilia and denied experiencing chest pain or respiratory difficulties. Given her recent surgery and presentation with vaginal bleeding, a transvaginal ultrasound was conducted, showing no retained products of conception.   

**Figure 1:**
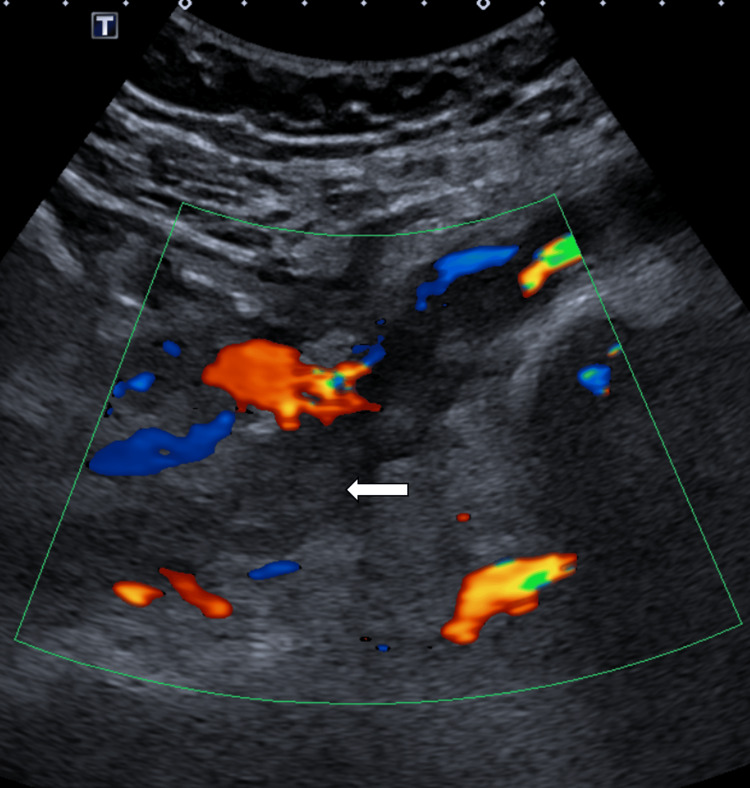
Duplex ultrasound of the left external iliac vein. The ultrasound shows a hyperechoic thrombus within the vein (white arrow) and an absence of flow within the vessel.

**Figure 2:**
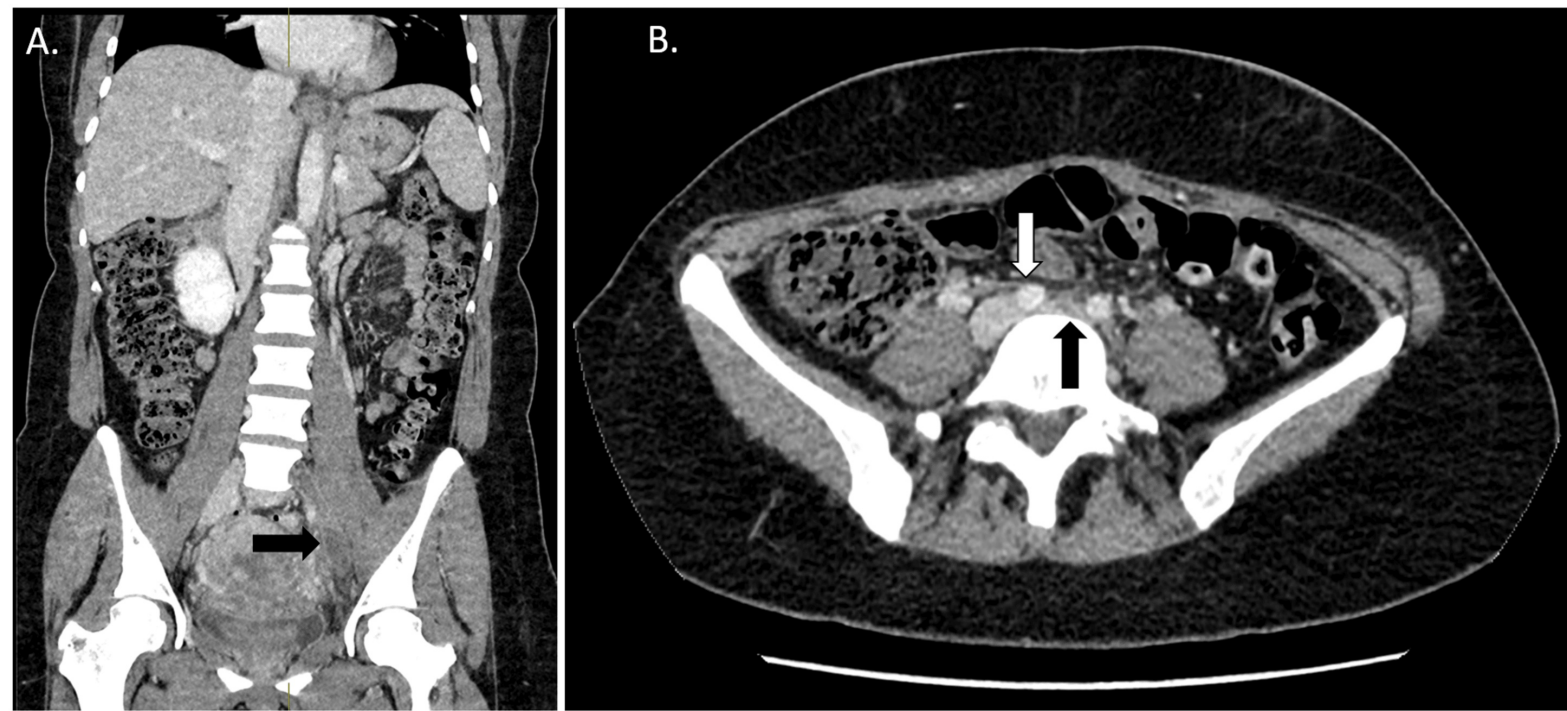
Iliofemoral deep vein thrombosis in the presence of May-Thurner syndrome. A. Coronal portal venous CT showing hypoattenuation within the left common and external iliac vein (black arrow). B. Axial portal venous CT, showing the overriding right common iliac artery (white arrow) and the compressed and thrombosed left common iliac vein (black arrow). CT, computed tomography; DVT, deep vein thrombosis

A venous duplex ultrasound of the left leg revealed absent Doppler flow and non-compressible left femoral and external iliac vein, in keeping with a complete occlusion (Figure 1). The proximal segment of the thrombus was not visualized on ultrasound, which necessitated a CT venogram; the CT confirmed an occlusive thrombus extending from the left common iliac vein to the left common femoral vein (Figure 2).  

Additionally, the CT venogram identified compression of the left common iliac vein by the right common iliac artery, indicative of May-Thurner syndrome, a structural abnormality predisposing individuals to iliofemoral DVTs due to chronic vascular compression (Figure 2) [13].  The patient was subsequently referred to interventional radiology (IR), where she successfully underwent PMT using the AngioJet™ system.  

Access was obtained via a left antegrade popliteal puncture, with a 9 French sheath and a 5 French, 100 cm straight-tip catheter. A 180 cm curved-tip 0.035” Terumo guidewire was used to cross the thrombus, after which the straight-tip catheter was exchanged for an 8 French, 105 cm Zelante AngioJet™ DVT catheter.  

**Figure 3:**
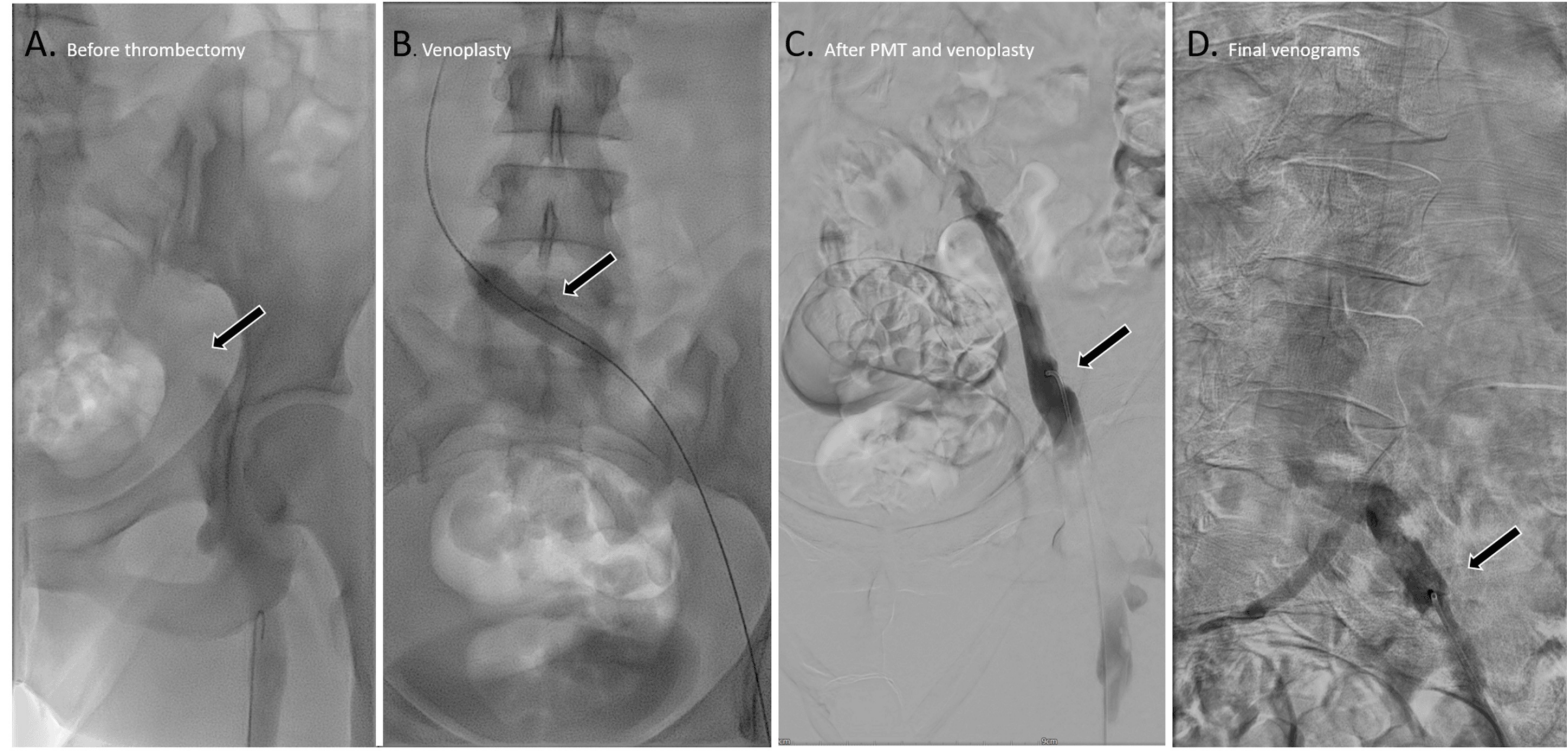
Venograms of the left common iliac vein, pre- and post-treatment. A. Radiograph showing a filling defect in the left common and external iliac vein (arrow). B. Radiograph showing balloon venoplasty of the left common iliac vein using a 12x40 mm balloon. C and D. Venogram showing recanalization of the left external iliac vein post-pharmacological and mechanical thrombectomy (arrow).

To facilitate thrombus lysis, 20 mg of Actilyse was injected directly into the clot. After 30 minutes, heparinized saline jets were used to fragment and aspirate the thrombus from the left iliac and femoral veins, requiring multiple passes due to the extensive clot burden. The post-thrombectomy angiogram confirmed successful revascularization, restoring patency to the occluded left iliofemoral veins (Figure 3).  

Following thrombectomy, venoplasty was performed using an Abbott Armada 35 (12x40 mm) plain balloon.  

Given the presence of May-Thurner syndrome, the patient was offered immediate venous stenting to reduce the risk of recurrent DVT; however, she declined the procedure.  

**Figure 4:**
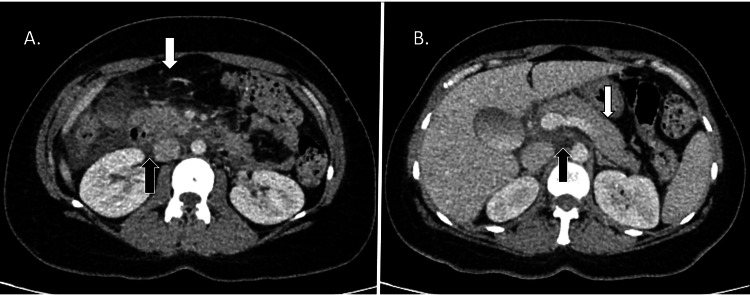
Contrast-enhanced CT of the abdomen showing acute pancreatitis. A. Axial cross-section of the mid-abdomen showing peripancreatic fat stranding (white arrow) and fluid in the hepatorenal pouch (black arrow). B. Axial cross-section of the upper abdomen showing bulky pancreatic body (white arrow) and bulky pancreatic head (black arrow). CT, computed tomography

The following day, she developed acute, severe epigastric pain. Laboratory tests indicated an amylase level of 1303 U/L (normal range: 28-100 U/L). These findings led to a clinical diagnosis of acute pancreatitis, further confirmed by a portal-venous phase CT of the abdomen showing significant peripancreatic fat stranding and bulky appearance of the pancreatic head, without evidence of biliary obstruction (Figure 4).  

The patient was managed conservatively by the general surgeons, which included intravenous hydration and analgesia. Her renal function remained within normal limits. Anticoagulation in this patient was challenging due to the recent miscarriage and ongoing vaginal bleeding, which worsened after PMT for a short period of time.  The patient’s hemoglobin level recorded two days post-procedure was 91 g/L (normal range 115-165 g/L). 

After fully recovering from both pancreatitis and the iliofemoral DVT, she was discharged with a scheduled follow-up outpatient appointment with the vascular surgeons to discuss the option of venous stenting of the left common iliac vein to mitigate the future risk of thrombosis due to May-Thurner syndrome.  

## Discussion

The AngioJet™  system is a type of PMT that combines pharmacological thrombolysis, through the infusion of a thrombolytic agent, with mechanical thrombectomy. Although most patients tolerate this procedure very well, it can rarely result in unfavorable complications; there have been occasional cases of acute interstitial pancreatitis reported in patients with comorbidities. This complication is thought to arise secondary to massive hemolysis, particularly in patients with chronic renal insufficiency [12,14].   

In a detailed case report by Danetz et al. (2004), two patients developed acute pancreatitis after undergoing mechanical thrombolysis with the AngioJet™  system. Both patients had different underlying conditions but shared common factors such as renal insufficiency and extensive thrombus burden, underscoring the pathophysiology linked to massive hemolysis during treatment [12].   

Another case, reported by Hershberger et al. (2011), involved a 53-year-old male who developed severe acute pancreatitis following percutaneous mechanical thrombectomy with the AngioJet™  system. The process in his case was thought to be related to extensive hemolysis triggered by the mechanical action of the thrombectomy, emphasizing the need for cautious operation of the procedure to avoid extensive hemolysis and associated complications. This case further supports the observation that extensive hemolysis can initiate an inflammatory process leading to pancreatitis [15].   

Pancreatitis secondary to hemolysis of various causes is well-documented. There is evidence from animal models that released heme groups may promote the activation of neutrophils, which have proteolytic and oxidative potential. This regulates the activation of alpha-2-macroglobulin (as an acute-phase reactant) and promotes intravascular coagulation, vascular permeability, and the generation of oxygen-free radicals in the microvasculature of the pancreas, mechanisms proposed to drive pancreatic inflammation [16].  A retrospective study of patients with massive hemolysis of various causes found acute pancreatitis occurred in over 20%, suggesting it may be an underrecognized complication [17].  

The risk of hemolysis is likely related to several factors, including the size of the thrombus, blood flow within the vessel, and the duration of the procedure. Symptoms may develop during the first 24 hours or even after four days post-procedure. Prevention strategies such as limiting procedural time, perioperative rehydration, and urine alkalinization may help prevent hemolysis-related complications, such as renal injury [18].  Urine alkalinization is achieved using medication such as sodium bicarbonate and helps to reduce the risk of renal injury by increasing the solubility of hematin, an acid produced by hemolysis [19]. 

The current literature demonstrates cases of acute pancreatitis in older patients, often with comorbidities such as renal failure. Our case report emphasizes the risk of acute pancreatitis following PMT in a young, healthy individual with normal renal function. We believe the contributing factors were likely due to dehydration and the maximum intensity and duration of the AngioJet™  system, leading to significant hemolysis. Although reducing the duration and intensity of PMT was not feasible in this case due to the substantial thrombus burden, generally minimizing these factors could help lower the risk of severe acute pancreatitis [20].   

Additionally, our patient had also undergone surgical treatment for a recent miscarriage. Despite normal renal function and hemoglobin levels, in hindsight, ensuring adequate hydration before and after the procedure appears to be a vital preventative measure for future interventions. 

## Conclusions

Percutaneous PMT using the AngioJet system is an effective treatment for acute iliofemoral DVT. 

However, it is not without risk, as hemolytic pancreatitis, although an exceedingly rare adverse effect, demonstrates the need to recognize acute pancreatitis as a significant, albeit uncommon, complication following the use of mechanical and pharmacological thrombectomy devices like AngioJet. This is particularly crucial in patients with predisposing factors such as renal insufficiency or extensive hemolysis during the procedure.  
